# Factors Influencing the Hydration, Dimensional Stability, and Strength Development of the OPC-CSA-Anhydrite Ternary System

**DOI:** 10.3390/ma14227001

**Published:** 2021-11-18

**Authors:** Zhenzhen Yang, Hansong Ye, Qiang Yuan, Baiyun Li, Yuelin Li, Dajun Zhou

**Affiliations:** 1 School of Civil Engineering, Central South University, Changsha 410075, China; yangzhenzhen1118@163.com; 2 National Engineering Laboratory for High Speed Railway Construction, Changsha 410075, China; 3 Zhejiang Communications Investment Group Co. Ltd., Hangzhou 310020, China; yehansong@cncico.com; 4 Foshan Transportation Science and Technology Co. Ltd., Foshan 528000, China; libaiyun1996@163.com; 5 Department of Civil Engineering, KU Leuven, Campus Bruges, 8200 Bruges, Belgium; yuelin.li@kuleuven.be; 6 ZhongNan Engineering Co. Ltd., Changsha 410075, China; zhoudajun@csu.edu.cn

**Keywords:** calcium sulphoaluminate, Portland cement, anhydrite, ternary binder, hydration, chemical shrinkage, drying shrinkage, compressive strength

## Abstract

Due to the advantages of high early strength and rapid setting, ternary systems consisting of ordinary Portland clinker (OPC), calcium sulphoaluminate (CSA) clinker, and anhydrite have broad application prospects. However, further studies need to be undertaken to find a more optimal mixing proportion of this ternary binder in order to meet basic performance requirements. In this paper, isothermal calorimetric tests, chemical shrinkage tests, drying shrinkage tests, and compressive strength tests were carried out to systematically identify the effect of the OPC/CSA ratio and anhydrite dosage on the hydration, mechanical property development, and dimensional stability of ternary binders. It was found that a higher CSA content leads to a higher cumulative hydration heat, a shorter acceleration period, and a delayed induction period, which can be ascribed to the retardation of C_3_S at a high aluminate concentration. However, a higher addition of anhydrite can retard the main peak of hydration despite promoting the intermediate peak and improving the hydration reaction rate. The drying shrinkage of blends decreases first along with the CSA proportion and then increases. Moreover, a higher anhydrite content mitigates the drying shrinkage and hinders the strength development. Finally, considering the properties of both the fresh and hardened binder, the ternary blends with 5% anhydrite and OPC/CSA ratios ranging from 3/7 to 2/8 were identified as most suitable for applications that require a high early strength, stable late strength, and small level of shrinkage.

## 1. Introduction

Ordinary Portland cement–calcium sulphoaluminate cement–anhydrite (OPC-CSA-CS) ternary composite binders have the characteristics of a high early strength, rapid setting [1], and adjustable rheological properties [2], which make them suitable for use in repairing mortars, shrinkage-free grouting materials, cement-based self-leveling screed [3,4,5,6], and 3D printing materials [7].

The performances of OPC-CSA-CS systems are governed by the calcium sulfate dosage and OPC/CSA ratio. The effect of calcium sulfate on hydration products has been well investigated in neat OPC or CSA systems. In the case of OPC, calcium sulfate reacts with C_3_A to form ettringite, which contributes to early strength development and improves the hydration degree [8]. Meanwhile. the adsorption of sulphate ions on C_3_A leads to blocking the active sites of dissolution and thus decreasing the dissolution rate of C_3_A [9], resulting in prolonging the setting time and delaying the OPC hydration. With the increase in the addition of sulphates, the cumulative hydration heat and heat peak first increase and then decrease [10]. A similar evolution can be observed in the compressive strength. The higher early strength is related to the acceleration of the alite reaction due to the retarding effect of sulphates on C_3_A. The reduced late strength is related to the increase in the S/Si and Ca/Si ratios and the presence of less water in the C-S-H [11]. Apart from this, the drying shrinkage also shows a trend of increase. This is because gypsum, the hydration product of anhydrite, is extremely easy to dehydrate and forms hemihydrate in regular environments [12]. Additionally, it should be noticed that delayed ettringite formation (DEF) can expand and cause damage to structures in the case of composite systems containing higher quantities of sulfates, for which the SO_3_/Al_2_O_3_ ratio can be used as an indicator [13]. Therefore, standards stipulate that the biggest dosage of sulfate in OPC should be no more than 3.5% [14].

For CSA, on the other hand, the addition of calcium sulphates affects the AFt/AFm ratio and water demand of complete hydration rather than modifying a variety of hydration products, such as AFm and AFt. [15]. The AFt/AFm ratio increases with the increase in the dosage of calcium sulfate. At a molar ratio of calcium sulfate to ye’elimite of 2, the quantity of ettringite reaches the maximum and the amount of monosulfate declines to zero [16]. Generally, ye’elimite (C_4_A_3_S), as the main phase of CSA, hydrates to form monosulfate and gibbsite (AH_3_). In the presence of sulphates, ettringite is produced [17] and they interlace with each other to form a network structure, contributing to a short setting time and high early strength [18]. This process is accompanied by the formation of AH_3_, which is amorphous or microcrystalline and contains a varying content of water [19]. In the early stage of the formation of ettringite, the induction period is shortened [20] and the hydration process of CSA is accelerated [21]. These effects mainly depend on the amount and reactivity of the calcium sulfates added [22,23]. Meanwhile, when the sulfate content is low, an AFm phase will be formed due to the conversion of ettringite. As the sulphate dosage increases, rapidly formed ettringite will block the contact between cement particles and calcium sulfates and water due to the surface coverage of cement grains and slow down the hydration rate, thus resulting in prolonging the setting time and decreasing the strength [18]. Moreover, the reduction in the generation of gel phases such as C-S-H and AH_3_ also causes a decrease in drying shrinkage with an increase in the sulphate content [24]. When the sulphate content is high, the stability of ettringite can be ensured so that it does not convert into AFm, which makes no obvious contribution to the strength development and does not expand. However, the excessive formation of ettringite often causes expansion, strength reduction, and even spalling. It has been reported that CSA does not expand when the sulfate dosage is lower than 30%, while an obvious expansion can be observed in conditions where the sulfate content is 50% [25], which can be understood through the hydrate phase variation with gypsum content shown by Glasser [26]. However, it should be noted that not all ettringite expands in these cases—this depends upon its morphology, growth site, nucleation rate, and formation time [27].

For OPC-CSA-CS ternary blends, the influences of the calcium sulfate dosage and OPC/CSA ratios on the hydration products and strength development have been investigated in some research works. With regard to the hydration products, it was reported that [1,28] when the OPC proportion in ternary composite systems is lower than 40%, C_4_A_3_S is given priority in the hydration process. In the absence of OPC, ettringite is formed by the reaction between C_4_A_3_S and calcium sulfates, as reported in Equation (1) [16].

(1) $$C_{4}A_{3}S+2\mathrm{CS}+38H\to C_{6}{\mathrm{AS}}_{3}H_{32}{+2\mathrm{AH}}_{3}$$

For another, in the presence of OPC, the formation of ettringite occurs due to the combination of C_4_A_3_S with calcium sulfates and CH, which is derived from the silicate reaction of OPC, as reported in Equation (2) [27].

(2) $$C_{4}A_{3}S+8\mathrm{CS}+6\mathrm{CH}+90H\to {3C}_{6}{\mathrm{AS}}_{3}H_{32}$$

Otherwise, it is dominated by silicate minerals. According to the literature, the liquid phase alkalinity after CSA hydration ranges from 10 to 11.5 [29], while that of OPC is approximately 13.5 [30]. Thus, we can say that a lower addition of OPC to ternary blends introduces silicate minerals that react to form CH and increase the pore solution alkalinity, which is beneficial for C_4_A_3_S hydration owing into reaction (2) [31]. Correspondingly, the incorporation of a small quantity of CSA to ternary blends introduces ye’elimite, which can react with CH, while its hydration product AH_3_ can consume CH to form ettringite as well, thereby decreasing the liquid phase alkalinity and promoting the hydration of C_3_S according to Le Châtelier’s principle [32]. Hence, the hydration of composite systems is a mutually promoting process. However, the issue of which mix can achieve the largest hydration degree and obtain the best performance has not yet been solved. In comparison with binary OPC-CSA blends, the addition of calcium sulphate promotes the hydration of ye’elimite. Whether forming AFt or monosulfoalmuminate, AH_3_ is a mutual product of the reaction between C_4_A_3_S and calcium sulphate and can further react with silicon reaction hydrates to form more AFt [33,34]. When calcium sulphate is consumed, the formation of Strätlingite—i.e., C_2_ASH_8_—results from the reaction of AH_3_ with calcium silicate.

In terms of strength development, Pelletier [35] studied the hydration process of an OPC-dominated ternary system whose sulphate content is within a range of 0 ~ 20% and found that there was a positive relationship between the AFt/AFm ratio and the compressive strength: the higher the calcium sulfate addition is, the higher the AFt/AFm ratio is. Due to the larger volume of AFt than AFm, an increased fraction of sulphates renders a higher volume of hydrates and a lower porosity, which leads to a higher compressive strength. However, Cui [36] investigated the influence of gypsum content ranging from 0 to 20% on the performance of ternary systems and found that a gypsum content higher than 13% led to the occurrence of a strength loss phenomenon. This is due to the formation of expansive hydrates and the decrease in active cement components in the case of high-sulfate content.

Regarding the dimensional stability, however, research works performed up to now have mainly focused on OPC-CSA binary systems. Yu et al. investigated the volume change of different OPC-CSA-CS compositions and concluded that an addition of 40% CS caused the expansion of ternary binders. Mixtures with a low quantity of CS were less likely to expand in spite of the increase in CSA, which can be attributed to the expansion failure caused by insufficient levels of CS and CSA [37]. The study conducted by Colonna [38] showed that the drying shrinkage of OPC-CSA blends reduces with the increase in the CSA mass ratio, which may be due to the fact that hydrated CH promotes the formation of more AFt. Saoût et al. [39] found that OPC-CSA blends have a similar chemical shrinkage to that of OPC in the first 40 h and that as more hydrates are generated, the chemical shrinkage of the composite paste becomes greater and its variation tendency presents a good agreement with hydration heat evolution [30]. Additionally, the chemical shrinkage increased with the increase in the amount of ettringite. By contrast, there was rather less effect on dimensional stability in ternary blends. Trauchessec [40] explored the effect of different anhydrite contents on the performance of ternary binders containing fixed OPC proportions and concluded that blends with 40% OPC, 46.5% CSA, and 13.5% anhydrite had optimal properties (in terms of strength and shrinkage).

Overall, the effects of calcium sulfate addition and OPC/CSA ratio on the early hydration and dimensional stability of ternary blends have not been well documented. Moreover, there is no agreement so far regarding what proportions of OPC, CSA, and CS will simultaneously grant ideal strength and dimensional stability. To fill these knowledge gaps, in this study the effects of calcium sulfate dosage and OPC/CSA ratios on the early hydration, chemical and dry shrinkage, and compressive strength of OPC-CSA-CS systems were experimentally investigated. Usable ternary blends were identified after removing specimens with obvious cracking or swelling. Moreover, attempts were made to identify OPC-CSA-CS binders with a high early strength, stable late strength development, and small shrinkage that may be suitable for industrial application. Based on these results, we proposed reasonable proportions of OPC, CSA, and CS for obtaining a balance between dimensional stability, strength, and fresh properties in OPC-CSA-CS systems.

## 2. Materials and Methods

### 2.1. Materials

**Figure 1:**
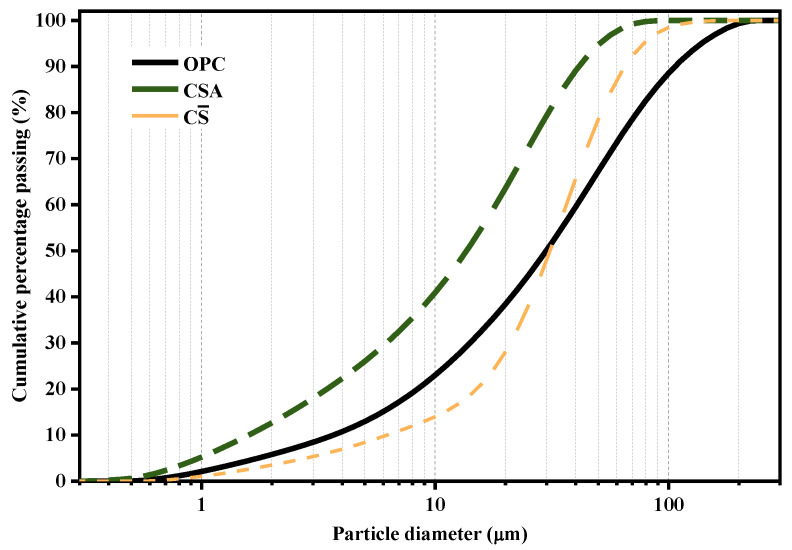
The particle size distributions of powder materials.

In this investigation, ordinary Portland clinker, calcium sulphoaluminate clinker, and anhydrite (China Building Materials Academy, Beijing, China) complied with the requirements of the Chinese standards GB/T 21372-2008, GB/T 37125-2018, and GB/T 9776-2008, respectively. Table 1 presents the chemical compositions and physical characteristics of cementitious materials. The particle size distributions of powdered materials were measured using the laser scattering technique and the test results are given in Figure 1. The mix proportions of the different OPC-CSA-CS ternary systems used in this study are shown in Table 2. For example, 1-9-5 represents an OPC/CSA ratio of 1:9 and a CS dosage of 5%. The proportion of OPC to CSA ranges from 1:9 to 9:1 under different CS contents (5 and 10%).

### 2.2. Specimen Preparation

The specimens used in this study were prepared as follows. Powder ingredients were first dry-mixed to achieve a homogenous state. The premixed powders were then added to a Hobart N50 mixer (Wuxi Construction Test Equipment Co., Ltd., Wuxi, China) and mixed with water for 60 s at a low speed, followed by resting for 60 s to allow the cleaning of the pastes attached to the blade and the mixer bowl. After that, mixing at low speed for 30 s was applied to minimize the amount of air trapped in the mixture.

### 2.3. Compressive Strength Test

A compressive strength test was carried out according to BS EN 196-1: 2016. After stirring, composite cement mortar was poured into 40 mm × 40 mm × 160 mm molds. Specimens were demolded after being cured in molds for 1 d and then cured at 20 ± 2 °C and a more than 90% relative humidity. The prism specimens were broken by flexure strength testing apparatus and then evaluated in a compressive strength test. The compressive strength of mortar cubes was then tested at 1, 3, 7, and 28-day.

### 2.4. Drying Shrinkage Test

Cement mortar specimens were prepared according to JC/T 603-2004. Specimens were demolded after 1 d and then cured in water for 1 d. The curing environment temperature was 20 ± 3 °C and the relative humidity was 50 ± 4%. The initial length (L_0_) of the specimens was measured by a comparator (Tianjin Beichen Construction Test Instrument Factory, Tianjin, China) and their length changes were recorded every day. The drying shrinkage rate can be calculated using Equation (1):

(3) $$S_{t}=\frac{\left(L_{0}-L_{t}\right)\times 100}{250},$$

where S_t_ (%) refers to the drying shrinkage rate of a specimen at age t, L_t_ (mm) refers to the specimen length at age t, and 250 (mm) is the effective length of the specimens.

### 2.5. Chemical Shrinkage Test

**Figure 2:**
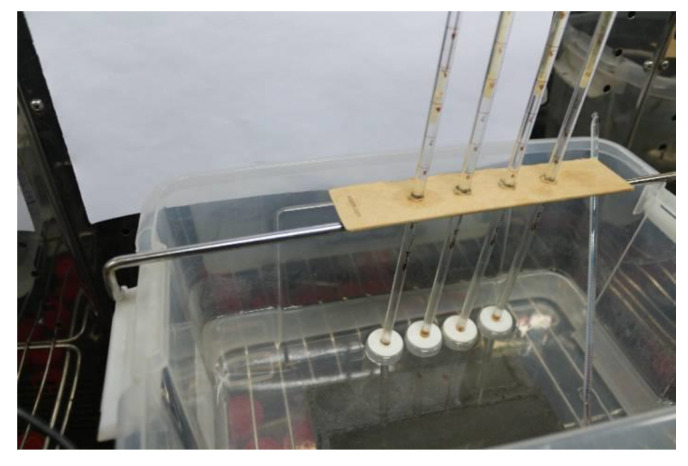
Photo of the chemical shrinkage test device.

The chemical shrinkage testing procedure was identical to ASTM C1608. Figure 2 shows the equipment used for the chemical shrinkage testing. Firstly, we filled the glass vial with an appropriate amount of fresh cement paste. It is best to cover the bottom of the vial with a 5 mm-thick layer. The vials needed to be tapped several times to minimize the amount of air trapped. Then, we slowly filled the glass vials with water without any agitation until it overflowed. We then inserted a rubber plug with a pipette into the vial and adjusted the water level in the pipette to a specified scale. Colored oil was injected into the tube with a long needle to reduce the moisture volatilization and enable it to be read conveniently. In order to prevent the water from overflowing during the testing process, sealant was used to seal the area of contact between the pipette and the rubber plug, as well as the contact point between the rubber plug and the bottle mouth. The equipment was placed in a constant-temperature water bath at 23 °C. Measurements started one hour after mixing.

### 2.6. Calorimetric Test

A TAM air thermal activity micro-calorimeter (TA instruments company, New Castle, DE, USA) with a twin-chamber testing channel was used to measure the hydration heat of ternary binders. One channel contained 7 g of distilled water and the other contained the same mass of blended cement paste, which was calculated according to the specific heat capacity. Throughout the test, the temperature surrounding the specimens was kept at 25 °C The test began 6 min after the contact of the cement and water. The data were recorded every ten seconds.

## 3. Results and Discussion

### 3.1. Hydration

**Figure 3:**
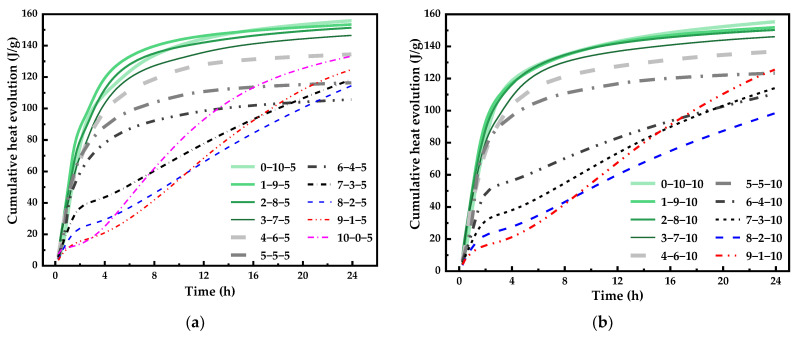
Cumulative hydration heat evolution of ternary blends with different CS contents: (**a**) with 5% CS; (**b**) with 10% CS.

Figure 3 presents the cumulative heat evolution of OPC-CSA-CS ternary binders with different CS contents. Only the hydration heat of the first 24 h is given, since the hydration process entered a stable period after 24 h. It can be seen that the use of a higher CS content in the OPC-CSA-CS mixtures had little effect on the cumulative heat released. For the CS contents of 5% and 10%, the cumulative heat released decreased from 155 J/g to around 105 J/g with an increase in the OPC/CSA ratio from 0/10 to 6/3, indicating a reduction in the hydration reaction degree [41]. Due to the formation of CH, the increased pore alkalinity promoted the hydration of C_4_A_3_S forming AFt and thus improving the hydration degree.

**Figure 4:**
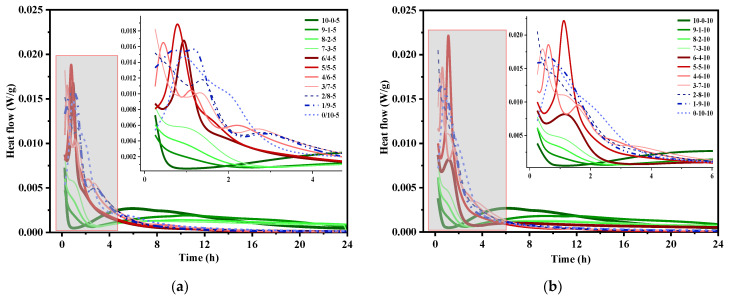
Hydration heat flow of OPC-CSA- CS ternary blends with different CS contents: (**a**) with 5% CS; (**b**) with 10% CS.

Figure 4 shows the effect of the OPC/CSA ratio and CS addition on the hydration heat flow of OPC-CSA-CS systems. In CSA-CS binary mixes, a higher CS content results in a higher hydration heat-release rate and a left-moving main heat peak, which indicates the acceleration effect of CS on CSA hydration [42,43]. CS in OPC-CSA-CS blends, however, retards the occurrence of hydration heat peaks, although the hydration heat-release rate is also improved. Additionally, it can be observed that there is no induction period in CSA-CS systems compared with OPC-CS blends [44]. Regarding ternary blends, when the aluminate ion concentration exceeds 2 mmol/L originating from CSA hydration, the hydration of C_3_S is suppressed, resulting in the induction period being prolonged [41]. Once the aluminate concentration remains lower than 1 mmol/l, the C_3_S hydration tends to accelerate. The delay of the C_3_S hydration may be associated with the poisoning of C-S-H nuclei by aluminate ions [45] or the condensation of alumino silicate and the formation of Si-O-Al bonds at the C_3_S surface [46]. Moreover, a smaller level of heat flow in the main hydration peak indicates the postponement of hydration by virtue of the incorporation of OPC in ternary binders. The main heat peak occurs later and the acceleration period is lengthened obviously when the OPC/CSA ratio increases. However, when the OPC/CSA ratio is higher than 5/5, the induction period cannot be well distinguished from the acceleration period. Apart from the exothermic main peak, an intermediate peak can be noticed between the initial peak and the second peak. As reported in [47], the intermediate peak is likely related to the formation of AFt/AFm or the dissolution of C_4_A_3_S. The intermediate peak occurs later as the OPC/CSA ratio increases from 1/9 to 5/5. Conversely, the higher the CS content is, the earlier the appearance of the intermediate peak is, and this can be ascribed to the reaction of AFt transforming into AFm in the absence of calcium sulfate [48].

### 3.2. Chemical Shrinkage

#### 3.2.1. OPC-CSA Binary Systems

**Figure 5:**
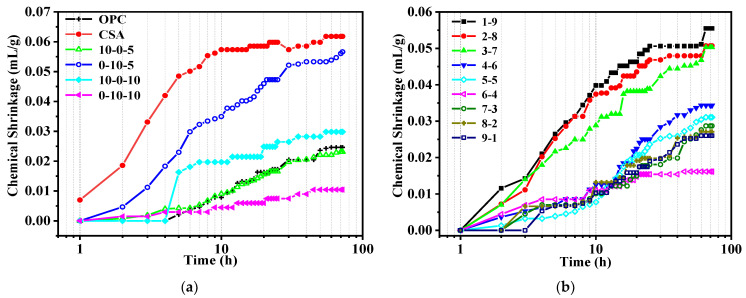
Chemical shrinkage of unitary systems and binary systems: (**a**) OPC or CSA or OPC-CS or CSA-CS blends; (**b**) OPC-CSA blends.

Figure 5a shows the effects of CS on the chemical shrinkage of unitary CSA or OPC systems. For the plain binder, the chemical shrinkage of CSA was almost 3 times higher than that of OPC because the formation of AFm requires more water than that of C-S-H. The incorporation of 5% CS showed little effect on the chemical shrinkage of both CSA and OPC systems, despite the 8% decrease within 72 h for the CSA system. With 10% CS, the chemical shrinkage of CSA was significantly mitigated by a reduction of 83% at 72 h and became around 50% lower than that of plain OPC. In contrast, 10% CS increased the chemical shrinkage of OPC from 0.025 to 0.030 mL/g after 72 h of hydration.

Figure 5b shows the chemical shrinkage of OPC-CSA binary systems with various OPC to CSA ratios. It was found that, with the increase in the percentage of OPC, the chemical shrinkage was first decreased and the lowest value was observed in the binder with an OPC to CSA ratio of 6:4, even lower than that of OPC. After that, the chemical shrinkage decreased again from the 7-3 group with the increasing in the proportion of OPC. Moreover, comparable chemical shrinkage values were observed for binders with a CSA content lower than 60%. Significant chemical shrinkage was observed in binders with a CSA content higher than 60%, where the chemical shrinkage values were almost doubled compared with those of other mixtures. Compared with neat OPC or CSA, composite cement consisting of a small amount of CSA and OPC or a small amount of OPC and CSA had a higher AFt formation rate and production [49] due to the mutual promotion effect. For mixes with high amounts of OPC and low amounts of CSA, the larger chemical shrinkage of C_4_A_3_S compared to silicates [50] causes an increase in chemical shrinkage with an increase in the proportion of OPC. However, in terms of binders with high CSA and OPC contents, the reduction in C_4_A_3_S is the reason for the lower chemical shrinkage despite the addition of OPC promoting C_4_A_3_S hydration [51,52]. The reason for the lower chemical shrinkage may be that plentiful AFt forms rapidly, creating skeletons that wrap unhydrated C_3_S and C_2_S [53] and thus delay the hydration process, indicating a lower level of chemical shrinkage. Moreover, the perturbing effect of high Al concentrations on C_3_S hydration is also responsible for the decrease in chemical shrinkage. Meanwhile, Yang et al. [54] found that the hydration of a mixture with 50% OPC and 50% CSA was greatly promoted, while excessive amounts of C-S-H gel were produced and deposited, which was not conducive to the growth of AFt. This also shows that the hydration mechanism is not been cleared out when the percentages of OPC and CSA are not very different.

#### 3.2.2. OPC-CSA-CS Ternary Systems

**Figure 6:**
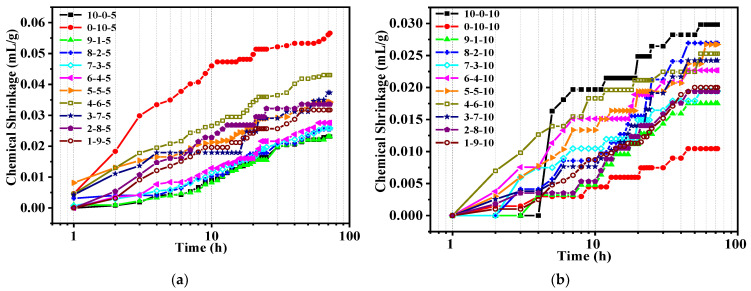
Chemical shrinkage of OPC-CSA-CS ternary systems: (**a**) with 5% CS; (**b**) with 10% CS.

Figure 6 shows the chemical shrinkage results of OPC-CSA-CS ternary systems with different OPC to CSA ratios and CS dosages. In the case of ternary systems with 5% CS (see Figure 6a), the chemical shrinkage increased with the increase in the proportion of CSA. An opposite trend, however, was noticed in ternary systems with 10% CS (see Figure 6b), where increasing CSA generally led to a lowering in the chemical shrinkage; the lowest value was found for the binder without OPC. The probable reason for the higher total amount of ettringite formed in this blend is the ettringite-formed reaction occurring between portlandite and aluminum hydroxide.

**Figure 7:**
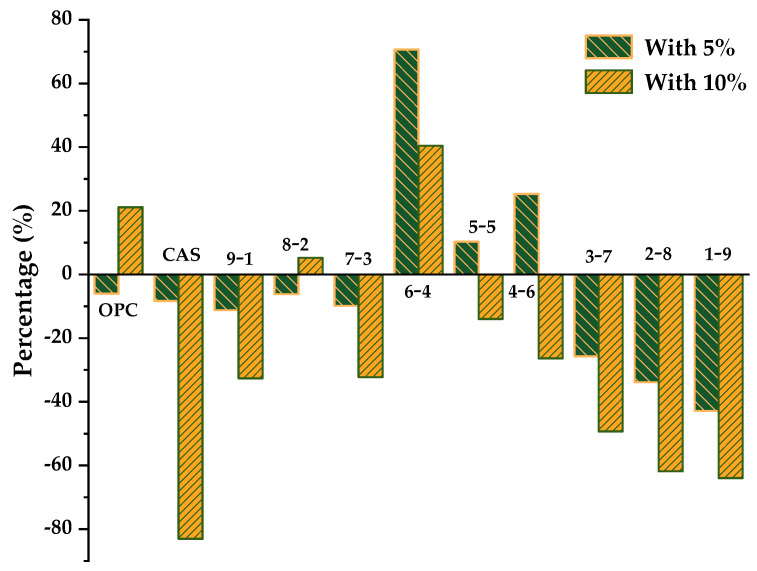
Comparison of chemical shrinkage of the OPC-CSA-CS and OPC-CSA systems at 72 h.

To better evaluate the influence of the addition of CS on the chemical shrinkage, the percentage was calculated by the difference between ternary blends and binary systems with the same OPC/CSA ratio based on the OPC-CSA system; the results are plotted in Figure 7. Positive values indicate an increase and negative values indicate a reduction. With the addition of 5% CS, the chemical shrinkage percentage of three groups, whose OPC/CSA ratios were 6/4, 5/5, and 4/6, respectively, showed a tendency to increase, while other groups showed a tendency to decrease. The largest increase of up to 70.6% appeared in the 6/4 ratio and the biggest reduction of up to 42.4% appeared in the 1/9 ratio. Likewise, for OPC-CSA-10%CS blends, the chemical shrinkage of most groups was decreased, except for the neat OPC, 8-2, and 4-6 systems. Additionally, the 6-4 group still displayed the largest rise in chemical shrinkage, while the pure CSA group showed the greatest reduction of up to 83%. Moreover, in the situation where there was a high CSA proportion (OPC/CSA < 5/5), the chemical shrinkage of ternary systems decreased with the decrease in the OPC/CSA ratio regardless of the CS dosage. By contrast, there was no obvious pattern of change in the case of a high OPC proportion.

**Figure 8:**
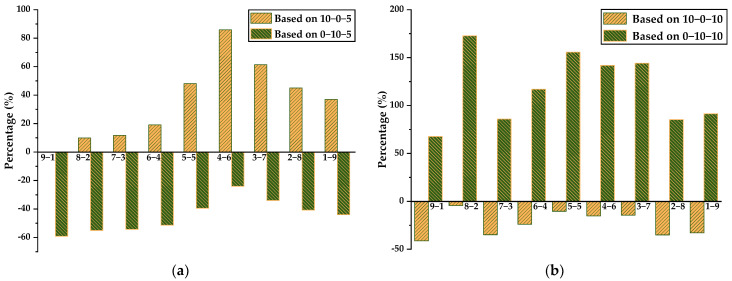
Chemical shrinkage of ternary systems and comparison: (**a**) with 5% CS; (**b**) with 10% CS.

Similarly, the effect of the OPC/CSA ratio on the chemical shrinkage of ternary binders at a fixed CS content is plotted in Figure 8. It can be seen that based on the OPC-CS blends, the incorporation of CSA promotes the chemical shrinkage of OPC-CSA-5% CS and causes the growth percentage to first increase and then decrease with the increase in the amount of CSA added. When taking CSA-CS blends as a reference, with the increase in OPC mass ratio, the percentage of reduction first decreased and then increased. A similar discussion could be conducted for the addition of 10% CS, where the opposite effect was observed.

### 3.3. Drying Shrinkage

#### 3.3.1. OPC-CSA Binary Systems

**Figure 9:**
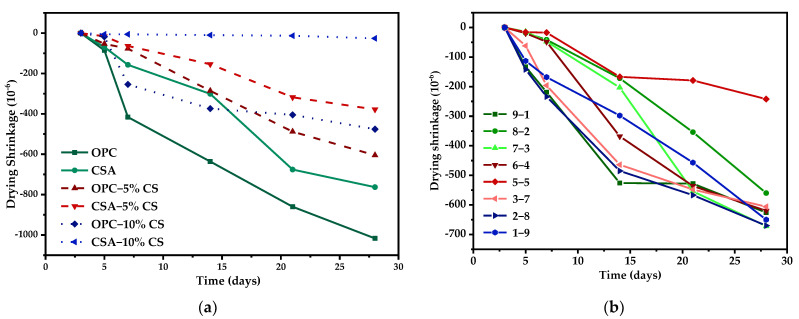
Drying shrinkage of unitary systems and binary systems: (**a**) OPC or CSA or OPC-CS or CSA-CS blends; (**b**) OPC-CSA blends.

Figure 9 shows the development of drying shrinkage for the binary systems. The drying shrinkage values of all binary systems increased over time, except for the CSA with 10% CS, which exhibited no obvious volume change. With regard to the effects of CS (Figure 9a), the addition of CS is conducive to reduce the drying shrinkage value for both unitary systems, and a better mitigation effect was observed in CSA than in OPC. For example, the drying shrinkage was reduced by 40.5% and 53.2% for OPC, while it decreased up to 99.6% for CSA. The addition of CS leads to the formation of ettringite with expansive properties instead of C-A-H or C-S-A-H gels, which show large levels of dry shrinkage due to water evaporation. This can explain why OPC-CS blends have a lower drying shrinkage than pure OPC paste. In contrast, a more significant decrease in the drying shrinkage of the CSA-CS system could be due to the balance between the dry shrinkage resulting from the hydration products of AH_3_ and the expansion raised by the growth of ettringite. On the one hand, the hydration of sulphoaluminate minerals is always accompanied by the formation of AH_3_, which is similar to C-A-H gel and able to cause drying shrinkage. On the other hand, the shrinkage can be compensated for by the formation of expensive ettringite, and the formation of ettringite can be promoted through the increasing addition of CS, as was reported in the work by Glasser et al. [26]. The best performance of the CSA with 10% CS could be attributed to its suitable nucleation and the growth rate of ettringite originating from the reaction between CS and sulphoaluminate mineral [25].

Figure 9b displays the effects of the proportions of OPC/CSA on the dry shrinkage in OPC-CSA binary systems. As shown in Figure 9b, with the increase in the CSA content, the dry shrinkage first decreased and then increased. The lowest dry shrinkage occurring at 28-day was observed in the specimen with 50% CSA, which had a nearly 50% lower shrinkage value when compared with other specimens.

#### 3.3.2. OPC-CSA-CS Ternary Systems

**Figure 10:**
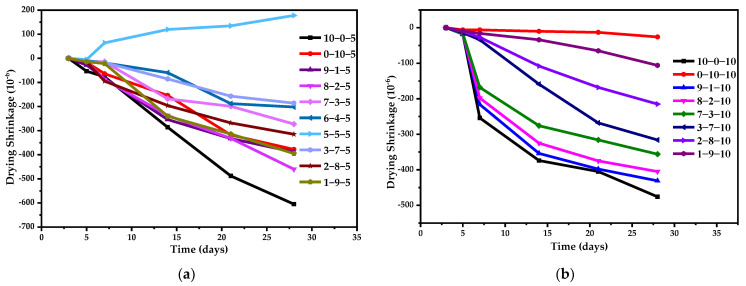
Drying shrinkage values of different pastes: (**a**) with 5% CS; (**b**) with 10% CS.

**Figure 11:**
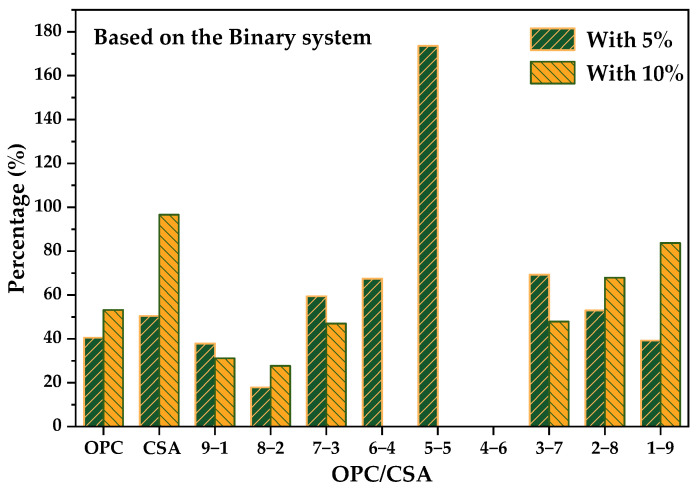
Effects of adding CS on the dry shrinkage of OPC-CSA binary systems.

The records of drying shrinkage values are exhibited in Figure 10. Several groups, such as 4-6-5, 6-4-10, 5-5-10, and 4-6-10, could not be measured due to fracturing that took place in the curing period. Figure 11 shows the effect of adding CS on the dry shrinkage of OPC-CSA binary systems. An example is given to illustrate the calculation method used. The percentage of 9-1-5 is equal to the difference between the drying shrinkage of the 9/1 binary system and that of 9-1-5 divided by drying shrinkage of the 9-1 binder. By comparing the results in Figure 10 with those in Figure 9b, it can be seen that adding CS reduced the dry shrinkage of OPC-CSA binary systems, and the 9-1 group with 10% CS showed the biggest dry shrinkage reduction of up to 83.7%. However, some cases, such as 5-5-5 group, exhibited swelling behavior with the incorporation of CS. This undesirable swelling could be attributed to the acceleration of C_4_A_3_S hydration caused by the addition of CS and the resulting rapid formation of ettringite at early hydration [55].

**Figure 12:**
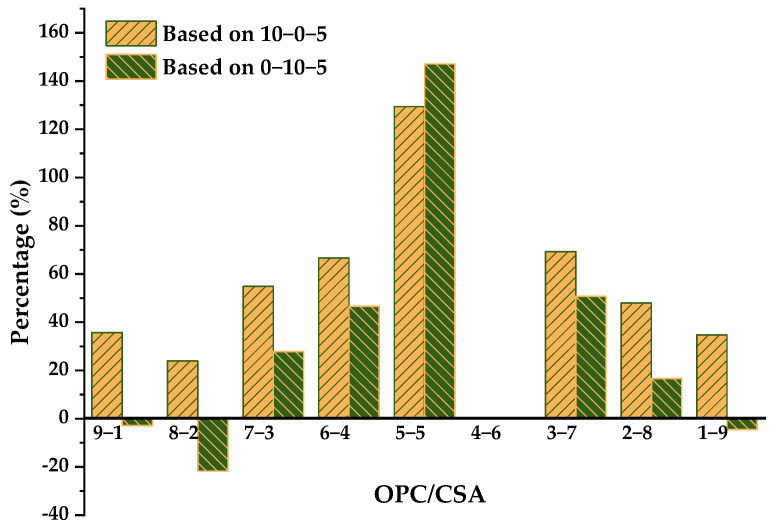
Drying shrinkage of ternary systems with 5% CS and comparison.

Figure 12 depicts the effect of adding CSA or OPC on the drying shrinkage of OPC-CS or CSA-CS binary systems. Due to the drying shrinkage of 0-10-10 being near to zero, a comparison cannot be made with the OPC-CSA-10% CS ternary systems. Based on the 10-0-5 group, which features the incorporation of CSA into OPC-CS binary binders, no matter how the OPC/CSA ratio changed, the drying shrinkage of composite systems decreased, among which that of 3-7-5 was mitigated the most. However, the drying shrinkage of three groups at OPC/CSA = 9/1, 8/2, and 1/9 was slightly larger than that of the 0-10-5 group.

### 3.4. Compressive Strength

#### 3.4.1. OPC-CSA Binary Systems

**Figure 13:**
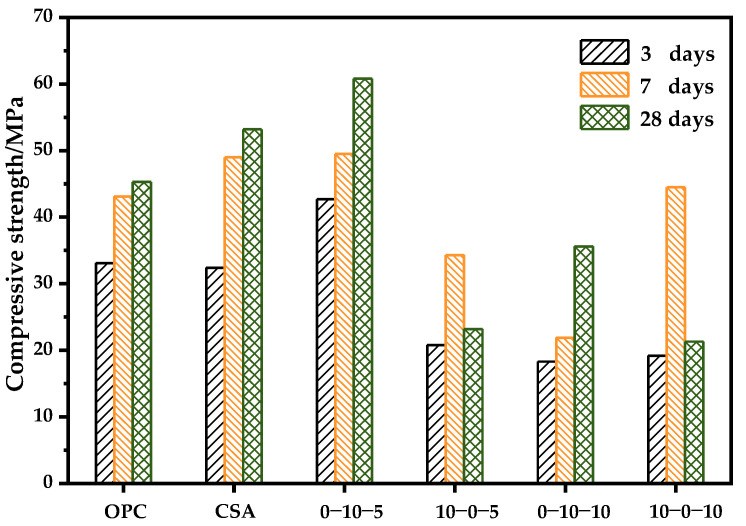
Effect of CS on the compressive strength of the single cement systems.

The effect of CS on the compressive strength of single cement is shown in Figure 13. It is clear that the CS content has a large influence on the compressive strength of both single systems. For single CSA, the addition of 5% CS boosts the compressive strength significantly at all tested age, while the addition of 10% CS plays an opposite role, which is consistent with the results reported in the literature [56]. A lower CS content can ensure the stability of the CSA strength, while a higher CS content can result in the excessive formation of ettringite, which may be the reason for the strength decrease seen for CSA-10% CS. However, for neat OPC the incorporation of CS at either 5 or 10% results in the appearance of a loss of strength at 28-day, and the strength at all tested ages declines to various degrees. The combined reaction of C_3_A and anhydrite causes ettringite to be generated slowly and continuously. A higher anhydrite addition leads to the over-saturation of ettringite, which can result in higher crystallization stresses that damage the microstructure and lead to strength loss or even cracking [57].

**Figure 14:**
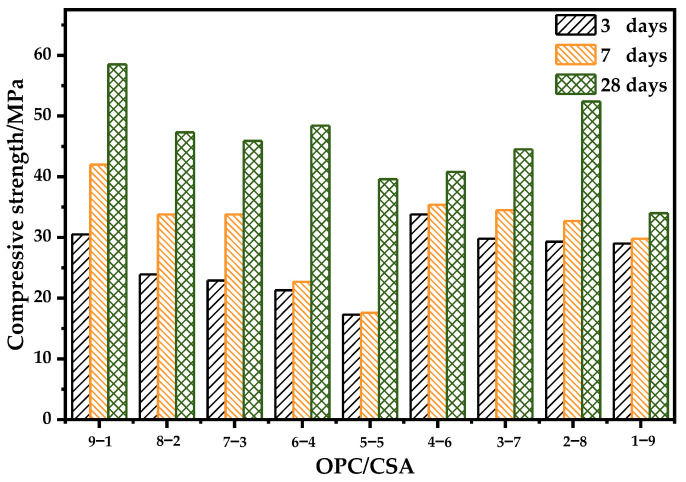
The compressive strength of OPC-CSA systems.

Figure 14 depicts the compressive strength of binary blends with different OPC/CSA ratios. The 3-day compressive strength decreases from approximately 31 MPa to 19 MPa with the increase in CSA content from 10% to 50%. A higher CSA content (>50%) rapidly increases the strength and maintains a value of around 30 MPa. In terms of the 28-day strength, the specimen with 10% CSA achieved the highest compressive strength, and the strength fluctuates between 40 and 50 MPa with the increase in CSA content up to 80%. The lowest strength of around 35 MPa is observed in the sample with 90% CSA. Overall, the results demonstrate that a CSA content ranging from 60 to 80% is conducive to simultaneously obtaining a higher early and late compressive strength. When the CSA addition is too high (i.e., the 1-9 group), ettringite due to CSA hydration forms a skeleton structure, while there is not enough gel to fill the pores between ettringites, which increases the early strength but limits the improvement of the compressive strength of the blends [43]. In contrast, when the OPC content is relatively high (i.e., the 8-2, 7-3, 6-4, and 5-5 groups), the strength development becomes slower with the increase in CSA. The primary factor influencing strength is the reaction degree. Due to the interference of aluminate ions in C-S-H nucleation, the addition of CSA retards the hydration C_3_S, as indicated in the heat evolution curves. The delay of C_3_S hydration at a high aluminate ion concentration can cause a slow strength development [41,58].

#### 3.4.2. OPC-CSA-CS Ternary Systems

**Figure 15:**
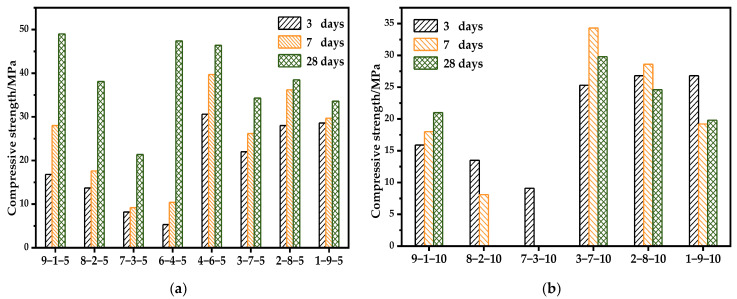
The compressive strength of OPC-CSA-CS ternary systems: (**a**) with 5% CS; (**b**) with 10% CS.

The compressive strength results of OPC-CSA-CS systems with two different dosages of CS are shown in Figure 15. It should be mentioned that some results for the OPC/CSA = 5/5, 6-4-10, 4-6-10, 7-3-10, and 8-2-10 groups could not be obtained because the failure of these specimens due to the swelling and cracking phenomena was observed in these samples before the target curing ages were reached.

Compared with the binary blends, the ternary blends show a similar strength variation trend with the change in the OPC/CSA ratio. It can be seen from Figure 15a that the compressive strength of ternary blends decreases with the reduction in the OPC/CSA mass ratio in the OPC-dominated systems when the dosage of CS is 5%. Among them, however, the compressive strength of the 6-4 group grows suddenly compared to that of the 7-3 group at 7-day and 28-day. When CSA becomes the main component, the compressive strength at 3-day shows substantial improvement compared with the OPC-dominated cementitious system, while the compressive strength at 28-day shows no significant growth in comparison with that at 3-day.

Figure 15b shows that when the dosage of CS is 10%, other experimental groups demonstrate a strength loss, except for OPC/CSA = 9/1, which may be related to the time inconsistency between the delayed ettringite formation and the setting and hardening of composite cement paste, resulting in the destruction of the microstructure and a reduction in its density [59]. The compressive strength decreases progressively with decreasing OPC content for the OPC-dominated cementitious system. Meanwhile, for the CSA-dominated cementitious system, the 7-day and 28-day strength decrease with the increasing CSA content and the 3-day strength is independent of the mass ratio of OPC/CSA. Furthermore, comparing two sets with the given mass ratio of OPC/CSA, it can be found that a higher C$\bar{S}$ dosage leads to a lower strength, especially with regard to the late strength.

### 3.5. Reasonable Value Range for the Ternary System Obtained Good Performance

Materials suitable for use in various applications should have a good fluidity and thixotropy, as small a shrinkage as possible, and be able to maintain a certain strength. Firstly, compressive strength deserves primary attention as a key embodiment of engineering application value. From the experimental results, it can be seen that a 10% CS content causes ternary systems to swell, leading to a decrease in strength, a loss of strength, or even a disappearance of strength. This finding also reflects the fact that a 10% CS content is not suitable if ternary blends need to be applied in engineering practice. In contrast, the series with 5% CS provided more favorable results in several mixes. For example, the mix with 10% CSA provided the highest 28-day compressive strength. Moreover, the mixes with a CSA content ranging from 60 to 90% exhibited a significantly enhanced early strength and a satisfactory 28-day strength. These mixes have great potential to be applied in practice.

The second most important aspect of performance is dimensional stability, which is strongly related to the durability and service life of a product. In general, it is desirable to keep shrinkage or expansion as small as possible in order to prevent cracks and any resulting degradation. On the one hand, compared with neat OPC and CSA as well as OPC-CSA binary blends, ternary blends generally have a lower drying shrinkage, except for a few mixes that exhibit swelling, which highlights the importance of the incorporation of CS. The results showed that a level of 10% CS is more efficient in controlling the dimensional stability, but this tends to cause poor compressive strength. Again, series with 5% CS are more favorable for use in practice, among which mixes with a CSA content of between 70 and 90% have more promising performances with regard to both their dimensional stability and compressive strength.

In addition to hardened properties, the rheological properties of fresh cement paste are also worth focusing on, and their relationships with the CS content and OPC/CSA mass ratio have been explored in a previous study. Details of this research can be found in our previous work [2]. It has been shown that OPC-CSA-CS ternary blends exhibit a shear-shinning behavior, which means that plastic viscosity decreases with an increase in the shearing rate, and this behavior contributes to good extrusion. A higher amount of CSA leads to a higher dynamic yield stress and apparent viscosity. However, an over-high yield stress and viscosity indicate the poor fluidity of pastes and are not conducive to their extrudability [60]. Furthermore, a higher CSA content increases the rate of structural build-up (the growth rate of static yield stress), indicating the enhancement of the resistance to bleeding and segregation as well as the resulting improvement in the quality of the interface between cement paste and aggregates [61]. Additionally, data obtained from isothermal calorimetry prove that the higher the proportion of CSA is, the faster the hydration rate is and the shorter the acceleration period is. That is to say that the time required for paste to set is shortened as well.

Consequently, the ternary systems selected to be the most suitable for use range from 3-7-5 to 2-8-5. These ternary combinations have the characteristics of a short setting time and good rheological properties (low dynamic yield stress and fast structural build-up rate), as well as favorable strength development (high early strength and stable late strength) and excellent dimensional stability (small shrinkage).

## 4. Conclusions

Basing on the above discussion, the following conclusions can be drawn:

(1) With a constant CS content, a higher CSA content results in a greater cumulative heat of hydration, indicating a higher reaction degree. When the OPC/CSA ratio is lower than 5/5, the existence of an intermediate peak can be observed, and this occurs later with an increasing OPC/CSA ratio for ternary systems. With the decrease in the OPC/CSA ratio, the hydration heat main peak occurs earlier and the acceleration period is shortened significantly, suggesting that the setting time is also shortened.

(2) With the increase in CSA, the chemical shrinkage of ternary blends containing small amounts of CS increases, while the chemical shrinkage of those with a high level of CS addition decreases, which may be ascribed to the perturbing effect of high Al concentrations on C_3_S hydration.

(3) For most OPC/CSA ratios, adding CS can reduce drying shrinkage. However, it is worth noting that mixes with an OPC/CSA ratio of around 1 tend to exhibit detrimental swelling, especially when a high CS content is added.

(4) In OPC-CSA-CS ternary blends, a CS content of 10% generally leads to a decrease in strength, a loss of strength, or swelling, which is caused by damage to the microstructure or cracking due to expansion generation, consequently limiting its use in practice. In contrast, the addition of 5% CS to OPC-CSA binders leads to a better strength development, especially in CSA-dominated systems.

(5) Considering the strength development, dimensional stability, rheological properties, and hydration, ternary blends with 5% CS and an OPC/CSA ratio ranging from 3 to 7 to 2 to 8 are proposed to be the most suitable for use in various applications.

## Figures and Tables

**Table 1:** Chemical compositions and physical properties of cementitious materials.

Cement	SiO_2_ (%)	Al_2_O_3_ (%)	Fe_2_O_3_ (%)	CaO (%)	MgO (%)	SO_3_ (%)	Other Oxides	Loss (%)	Density (g/cm^3^)	Blaine (cm^2^/g)
OPC	20.76	4.58	3.27	62.13	3.13	2.8	1.21	2.12	3.14	2845
CSA	11.72	22.83	1.50	46.79	1.72	12.09	2.02	1.33	2.81	3770
CS	3.03	0.59	0.29	39.53	0.68	54.62	0.69	0.57	2.97	3840

**Table 2:** The mix proportions of different OPC-CSA-CS ternary composite systems.

Specimens	CS (%)	OPC: CSA	w/b Ratio	Remarks
1-9-5	5	1:9	0.5	The ratio of OPC:CSA and dosage of CS in other ternary composite systems follow similar rules to those of the examples.
9-1-5	5	9:1	0.5	
2-8-10	10	2:8	0.5	
8-2-10	10	8:2	0.5	

## Data Availability

The data presented in this study are available on request from the corresponding author.
